# A commentary on “Autofluorescence alone is not enough: a combined strategy for accurate identification of parathyroid tissue in thyroidectomy specimens”

**DOI:** 10.1097/JS9.0000000000004728

**Published:** 2026-01-12

**Authors:** Jia-Hao Cao, Wei Li, Bo Zhang

**Affiliations:** a Department of Thyroid Surgery, The Second Affiliated Hospital of Jiaxing University, Jiaxing, China; b Department of clinical laboratory, The Affiliated Hospital of Jiaxing University, Jiaxing, China

*Dear Editor,*

We read with great interest the study by Kuo *et al*[1] demonstrating that combining near-infrared autofluorescence (NIRAF) imaging with tissue parathyroid hormone (PTH) washout testing significantly improves the diagnostic accuracy of identifying inadvertently excised parathyroid glands. Although their prospective cohort study provided compelling evidence for this multimodal approach, we believe that it confused the disparate concepts of “detection” and “preservation,” thereby potentially overstating the clinical value of this technique and obscuring more fundamental issues in the strategy of parathyroid protection.

In this study, “inadvertently resected parathyroid gland” was clearly defined as physically resected tissue in thyroidectomy specimens, and the detection rate of postoperative NIRAF scanning was 8.7%. The conclusion of this study indicates that this technique may contribute to the preservation of parathyroid function. However, it primarily focuses on glands that have been surgically removed while overlooking vascular damage to the remaining parathyroid glands, which represents another critical factor contributing to postoperative hypoparathyroidism. Parathyroid function depends on the integrity of microvessels. Studies have shown that the loss of parathyroid function is more due to the destruction of its fragile blood supply than to the unintentional physical removal[2]. The study’s impressive 100% detection rate for excised glands does not translate to 100% prevention of functional loss, because most functional loss occurs through vascular compromise – an injury NIRAF cannot detect. This creates a false causality: the reported 0.4% permanent hypoparathyroidism rate likely reflects the senior surgeons’ inherent skill in preserving blood supply during dissection, not the remedial impact of NIRAF salvage. In essence, the study proves NIRAF can find glands in a specimen basin, but presents this as “preservation,” conflating a *post hoc* rescue maneuver with intraoperative prevention, and thereby overpromising clinical utility for a problem it does not actually solve. Critically, the study does not correlate NIRAF findings with intraoperative vascularity assessment (e.g., indocyanine green angiography), which has been shown to identify poorly perfused PTGs^[3,4]^. Without perfusion data, the claim that NIRAF “supports more informed parathyroid preservation” is premature. A gland that is NIRAF-negative in the specimen may have been left devitalized in the neck – the outcome that truly matters for patients.

Beyond this conceptual error, the rigid application of a ≥100 pg/ml PTH cutoff for autotransplantation constitutes a binary oversimplification of parathyroid gland viability. PTH secretion is exquisitely sensitive to intraoperative variables, including ischemia time, mechanical trauma, and temperature fluctuations^[5,6]^. Consequently, a single intraoperative PTH value is an inadequate surrogate for functional potential. This limitation is illustrated by the study’s methodology, which discarded 27 autofluorescence-positive tissues with PTH levels below the threshold, including two that were histologically confirmed as parathyroid glands – thereby representing potential false-negative exclusions based on an arbitrary metric. Feasibility assessment requires a more nuanced dynamic approach, including metrics such as the decay rate of PTH concentration in serial sampling, rather than relying on a single static measurement. By treating parathyroid tissue as a static diagnostic entity, the authors’ protocol fails to account for the variable regenerative capacity inherent to a living graft.

The diagnostic accuracy metrics may be inflated by incorporation bias. NIRAF-positive areas underwent more intensive histopathological examination than NIRAF-negative lobes, which were only “carefully examined” without systematic random sampling. True reference standard validation requires blinded, protocolized sectioning of entire specimens, regardless of fluorescence status. Without this, the reported 97.9% specificity may overestimate performance in unselected practice where pathologists lack similar vigilance.

We propose that parathyroid protection requires a three-domain assessment: (1) Detection (NIRAF), (2) Perfusion (e.g., indocyanine green angiography), and (3) Functional Reserve (dynamic PTH testing)[7]. Only by integrating these modalities can surgeons distinguish between a healthy in situ gland, a devascularized gland requiring autotransplantation, and an exhausted graft unlikely to function. The authors’ study provides valuable data for the first domain but stops short of addressing the true determinants of parathyroid insufficiency. Future trials must include perfusion assessment and long-term graft function to determine whether this combined strategy meaningfully reduces morbidity beyond what expert surgeons already achieve.

## Data Availability

Not applicable.
